# Optimizing Osteogenic Differentiation of Ovine Adipose-Derived Stem Cells by Osteogenic Induction Medium and FGFb, BMP2, or NELL1 In Vitro

**DOI:** 10.1155/2018/9781393

**Published:** 2018-09-26

**Authors:** Emil Østergaard Nielsen, Li Chen, Jonas Overgaard Hansen, Matilda Degn, Søren Overgaard, Ming Ding

**Affiliations:** ^1^Orthopaedic Research Laboratory, Department of Orthopaedic Surgery and Traumatology, Odense University Hospital, Department of Clinical Research, University of Southern Denmark, Sdr. Boulevard 29, 5000 Odense, Denmark; ^2^Department of Endocrinology and Metabolism, Molecular Endocrinology Laboratory (KMEB), Odense University Hospital, University of Southern Denmark, J. B. Winsløws Vej 25.1, 5000 Odense, Denmark; ^3^Department of Pediatrics and Adolescent Medicine, University of Copenhagen, Rigshospitalet, Blegdamsvej 9, 2100 Copenhagen, Denmark

## Abstract

Although adipose-derived stromal cells (ADSCs) have been a major focus as an alternative to autologous bone graft in orthopedic surgery, bone formation potential of ADSCs is not well known and cytokines as osteogenic inducers on ADSCs are being investigated. This study aimed at isolating ADSCs from ovine adipose tissue (AT) and optimizing osteogenic differentiation of ovine ADSCs (oADSC) by culture medium and growth factors. Four AT samples were harvested from two female ovine (Texel/Gotland breed), and oADSCs were isolated and analyzed by flow cytometry for surface markers CD29, CD44, CD31, and CD45. Osteogenic differentiation was made in vitro by seeding oADSCs in osteogenic induction medium (OIM) containing fibroblast growth factor basic (FGFb), bone morphogenetic protein 2 (BMP2), or NEL-like molecule 1 (NELL1) in 4 different dosages (1, 10, 50, and 100 ng/ml, respectively). Basic medium (DMEM) was used as control. Analysis was made after 14 days by Alizarin red staining (ARS) and quantification. This study successfully harvested AT from ovine and verified isolated cells for minimal criteria for adipose stromal cells which suggests a feasible method for isolation of oADSCs. OIM showed significantly higher ARS to basic medium, and FGFb 10 ng/ml revealed significantly higher ARS to OIM alone after 14 days.

## 1. Introduction

Several conditions such as trauma, tumor, infection, and surgical procedure can cause larger bone defects. Due to the lack of easily accessible new bone formation materials, patients with these problems can be faced with major clinical challenges that affect treatment. Autograft primarily harvested from the iliac crest of the same patient is the gold standard as new bone formation material. Autograft bears the fundamental characteristics for new bone formation: osteogenesis, osteoinduction, and osteoconduction [1]. Nonetheless, harvesting autograft has its disadvantages and complication frequency of between 8.5% and 20% has been reported. Complications from harvesting this material include infections, chronic pain, blood loss, and fractures from the donor site [2], and an important limitation is the restricted amount of autograft available for harvesting [3].

Mesenchymal stromal cells (MSC) as progenitor cells have been investigated regarding their capability to generate new bone tissue. These cells have displayed promising results and have the potential to replace autograft because of its good proliferation and osteogenic properties [4]. The most investigated MSC is the bone marrow-derived multipotent mesenchymal stromal cells (BMSCs) which have shown the most interesting results regarding new bone formation in vivo [5]. BMSCs are already being tested in preclinical [6] and clinical studies [7]. The disadvantages of this method are a low concentration of MSC in bone marrow aspirate, discomfort, and morbidity for the patient [8].

Adipose-derived stromal cells (ADSCs) have been investigated because they have the same properties as BMSCs. Easy access to the adipose tissue (AT) as well as the amount of this tissue in the human body together with high stem cell (SC) counts makes it an interesting area to explore [9]; moreover, ADSCs are easier to harvest when compared to BMSCs and have a lower risk of complications [8, 10]. It is important to ensure that your data is translatable to other studies; therefore, a minimal criteria for adipose stromal cells (ASCs) proposed by the Federation for Adipose Therapeutics (IFATS) and the International Society for Cellular Therapy (ISCT) was made by Bourin et al. [11].

Preclinical trials in large animals with ADSCs are necessary to obtain morphological and biomechanical information on bone repair before clinical trials [12]. Our recent study comparing cells derived from ovine bone marrow (BM) and cells from ovine AT revealed that the BM has superior ability to form new bone in vivo compared to AT in a severe combined immunodeficiency mouse (SCID) model [13] which is in line with recent studies comparing BMSCs and ADSCs [14–17]. Although new bone formation was seen in both AT and BM groups, the quantitative histomorphometry showed that the bone formation in the AT groups was 10-fold lower than in the BM groups [13].

Many factors may influence bone formation, and a key factor might be the osteogenic differentiation and commitment to osteogenic lineage. As summarized below, several known growth factor cytokines have been tried for osteogenic differentiation both in vitro and in vivo. To our knowledge, no studies have compared more than two growth factors on ADSCs in vitro and later in vivo.

Recombinant human fibroblast growth factor basic/2 (FGFb) has been shown to have positive osteogenic effects on human BMSCs and human ADMSCs by enhancing osteogenic differentiation in vitro and bone osteoid area in vivo [15]. Little is known about FGFb and its influence on ovine bone formation [18]. No studies have tested FGFb on any kind of ovine progenitor cells although FGFb has shown better bone formation in smaller animal models [19].

Bone morphogenetic proteins belong to the transforming growth factor-*β* family which is the best investigated enhancer of bone genesis. Recombinant human bone morphogenetic protein 2 (BMP2) has been widely studied and has been indicated to have great potential in bone formation in vitro and in vivo [20, 21]. Today, it is also used in clinical applications [22]. BMP2 has been shown to enhance results on human ADSC osteoblastic phenotype [21, 23] and in ovine ADSCs (oADSCs) in nonunion of the tibia [12]. BMP2 has also been shown a dose-dependent adipogenesis activation through peroxisome proliferator-activated receptor *γ* (PPAR*γ*) [24]. The effect of BMP2 in vivo is not well known and is probably highly dependent on the environment of applications suggested by Kim and Choe [25].

Recombinant human NEL-like molecule 1 (NELL1) is a secretory glycoprotein first discovered through its association with craniosynostosis (CS) in children [26]. Transgenic mice overexpressing NELL1 have also shown an association with CS [27], and mice with a deficiency of the of NELL1 gene have shown defects in calvaria and long bone volume which suggests that NELL1 may have an important role in bone development [28, 29]. The direct cellular role of NELL1 is not well known but is believed to have a downstream Runt-related transcription factor-2 (Runx2) activation separated from BMP2's signaling pathway [30]. It has been shown that NELL1 downregulates the expression of PPAR*γ* and has an antiadipose pathway regulation; thus, a combination of NELL1 and BMP2 may lead to enhanced osteogenesis and have antiadipose tissue regulation [31]. NELL1 has been shown to boost the bone formation in osteoporotic ovine in the surrounding tissue of administration which suggests that the human version of the protein has a similar role in ovine [29].

This study investigates osteogenic differentiation on oADSCs by optimizing bone engineering (harvesting, seeding, and culturing) in vitro. We use Alizarin red staining (ARS) for calcium deposit in the extracellular matrix (ECM) of oADSCs which is believed to have high sensitivity for osteogenic differentiation [32, 33]. These results may possibly lead to more promising use of ADSCs in today's preclinic and clinic.

The primary aim of the study is to isolate oADSCs and verify for minimal criteria for ASCs. The secondary aim is to optimize the conditions for oADSC osteogenic differentiation by medium and growth factors.

We hypothesize that adding more bioactive factors to growth medium will improve the commitment to osteogenic differentiation of oADSCs which may significantly enhance the commitment to osteogenic lineage.

## 2. Method and Materials

### 2.1. Isolation and Expansion of Stem Cells from Adipose Tissue

Surgical procedures were performed at the Biomedical Laboratory at the University of Southern Denmark. A total of *n* = 4 AT extractions were made on 2 experimental female sheep (Texel/Gotland breed, 2–4 years of age) lateral to the vertebra on both sides (average sample weight of 6.58 g) under local anesthesia with 5 ml of lidocaine s.c. (Amgros, Copenhagen, Denmark), systemic Rompun (1.0–1.2 ml Vet, Bayer, Germany), and Temgesic (0.6–0.7 ml, Reckitt Benckiser, Hull, UK).

After scalpel incision, the sample was placed directly in falcon tubes with 5 ml of preheated (37°C) Dulbecco's phosphate-buffered saline (PBS, Gibco, cat. no. 14040-091, Roskilde, Denmark) with 10% bovine serum albumin (BSA; Sigma Aldrich, Denmark) and 1% penicillin/streptomycin (P/S; Sigma Aldrich). The oADSCs were obtained according to Gimble [34]. Samples were immediately taken to the cell culture lab where they were put into petri dishes and washed with PBS and 1% P/S. The samples were then put in falcon tubes, and 200 units/ml collagenase type I (Thermo Fisher, cat. no. 17018-029, Denmark) were added to them (10% BSA, 1% P/S in scale 1 : 1 (volume : weight)). Afterwards, the tubes were placed in a 37°C water bath for 60 min with shaking after which they were centrifuged at 300g for 5 minutes. This was followed by the careful aspiration of the adipocyte layer and liquid to isolate stromal vascular fraction (SVF) pellets. 1 : 10 erythrocyte lysis buffer (ELB, BD, cat. no. 555899, Denmark) in sterile water was then added and incubated at room temperature (RT) for 10 minutes. After incubation, cells were filtered through 100 *μ*m filter to get rid of cell debris.

The harvested cells were centrifuged at 300g for 5 minutes after which the supernatant was discarded and the SVF pellets resolved in Dulbecco's modified eagle's medium/nutrient F-12/GlutaMax (DMEM, cat. no. 31331-028, Gibco) with 10% fetal bovine serum (FBS, Gibco, cat. no. 10270-098) and 1% P/S (hereafter referred to as *basic medium*). Cells were then mixed by pipet and counted by hemocytometer after which they were seeded in T75-150 flasks in basic medium dependent on SVF isolation yield and cultured in incubator 37°C with 5% CO_2_ for 6–9 days before cell attachment was observed. Medium was then changed to wash away nonadherent cells, and the remaining cells were expanded until 60–85% confluence was reached. Trypsin/ethylenediaminetetraacetic acid (EDTA, Sigma Aldrich) was used to transfer cells for future further culturing.

### 2.2. Colonizing Forming Unit (CFU) Assay

**Figure 1:**
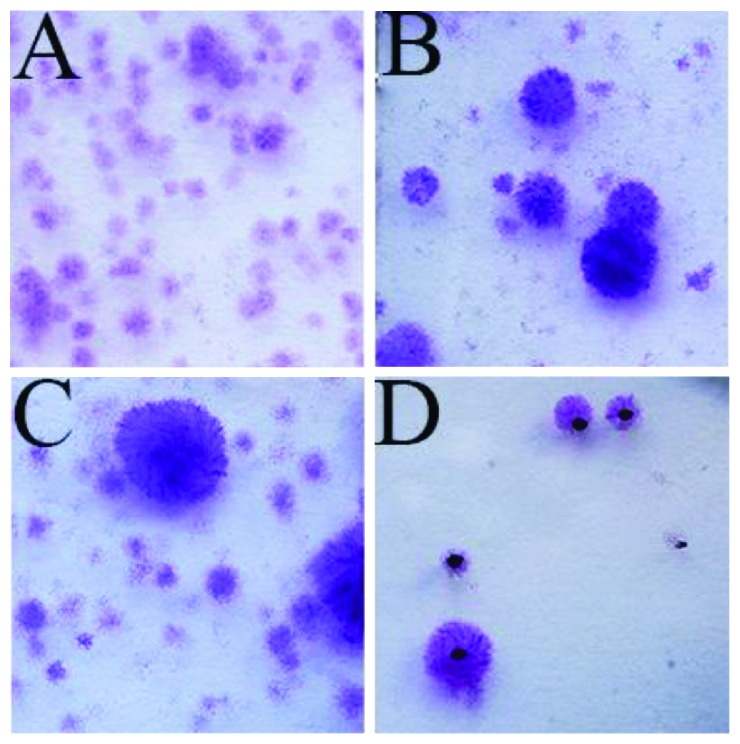
Part of the colonizing forming unit assay made by the crystal violet methanol after seeding SVF cells in T75 flasks. (A) Seeding of 1·10^5^ SVF cells from sample 1 for 7 days. (B) Seeding of 1·10^4^ SVF cells from sample 2 for 12 days. (C) Seeding of 1·10^4^ SVF cells from sample 3 for 12 days. (D) Seeding of 1·10^3^ SVF cells from sample 2 for 12 days. Low density of CFU is seen, and black dots form counting.

CFU assays were made on SVF samples to measure proportion of ADSCs in the SVF isolation [35]. From sample one, 1·10^5^ cells from SVF were seeded in a T75 flask. These cells were cultured for 7 days and analysed after standard protocol. In brief, cells were washed twice with PBS, fixed by 4% paraformaldehyde (PFA), washed in PBS, stained with 0.5% crystal violet methanol for 30 minutes, and finally washed with water to remove nonspecific staining. CFU were counted positive if more than 50 cells in a colony were observed. Due to the high number of CFU in the first sample, later SVF samples were seeded in lower numbers (1·10^4^ and 1·10^3^ cells per T75 flask) for 12 days to make counting easier (Figure 1) and more reliable like the original protocol by Castro-Malaspina et al. [36].

### 2.3. Flow Cytometry (FC) Analysis

ADSCs from passage 1 (P1) were cryopreserved by standard protocol in 10% dimethyl sulfoxide (DSMO, Sigma Aldrich, cat. no. C6295) and kept at −80°C. After thawing, the cells were grown one passage. P2 cells were harvested and analysed for the expression of surface antigens by staining with CD29-APC (BioLegend, cat. no. 303007, clone TS2/16), CD45-PE (Bio-Rad, cat. no. MCA2220PE, clone 1.11.32), CD44-FITC (Bio-Rad, cat. no. MCA2219F, clone 25.32), or CD31-FITC (Bio-Rad, cat. no. MCA1097F, clone CO.3E1D4) for FC analysis on a FACSVerse (BD Biosciences). Corresponding isotype antibodies from BD Pharmingen were used as negative controls. In brief, cells were detached by EDTA, washed with PBS, counted, and stained with fixable viability dye eFluor 506 (eBioscience, cat. no. 65-0866-14) to show live/dead cells. The cells were subsequently fixed by cytoperm/cytofix (BD, cat. no. 51-2090KZ). 5·10^5^ cells in each sample were washed with PBS and stained with relevant antibodies for 30 min at 4°C in the dark. The cells were stained for CD45 and CD29 in addition to either CD44-FITC or CD31-FITC, and another tube was stained with the isotype controls. 5–10 *μ*l antibody solution per 200 *μ*l cell suspension was used according to the manufacturer's recommendations.

### 2.4. Osteogenic Differentiation

oADSCs from P1 were resuspended in basic medium and counted. 4,000 cells/cm^2^ were seeded in 4 flat-bottom 24-well plate with no coating (DACOS, Denmark), and 1 ml basic medium was added to each well. After 24 hours in a 37°C incubator, the 5% CO_2_ medium was replaced with six different growth mediums. Each of the four plates had a different dosage (100 ng/ml, 50 ng/ml, 10 ng/ml, and 1 ng/ml) of growth factors:

1. Basic medium (control)
2. Basic medium with osteogenic induction medium (OIM) consisting of L-ascorbic 0.2 mM (Wako, cat. no. 013-12061, USA), dexamethasone 1·10^−8^ M (Sigma, cat. no. D4902), and NaH_2_PO_4_ 3 mM
3. Basic medium, OIM, and rhFGF-basic (FGFb) (R&D, cat. no. 233-FB-025, Minneapolis, USA)
4. Basic medium, OIM, and rhBMP2 (BMP2) (R&D, cat. no. 355-BEC-010, Minneapolis, USA)
5. Basic medium, OIM, and rhNELL1 (NELL1) (R&D, cat. no. 5487-NL-050, Minneapolis, USA)
6. Basic medium, OIM, rhBMP2, and rhNELL1

In each well, the medium was changed every 48–72 hours for 14 days. Medium was freshly prepared each time to minimise protein decay, and growth factors were added just before use. Technical triplicates were made in all groups.

### 2.5. Alizarin Red Staining and Quantification

After 14 days of osteogenic differentiation, medium was removed, and cells were both washed by PBS and fixed by 70% ethanol for 1 hour at −20°C. Afterwards, the cells were briefly washed in dH_2_O. 40 mM Alizarin red (Sigma Aldrich, cat. no. A-5533) pH was adjusted to 4.1-4.2 and was added and placed under rotation for 10 minutes. Dye was removed and washed by dH_2_O twice. PBS was added and placed under rotation for 5 minutes to wash away nonspecific stains. Pictures of stained layers were taken by scanner and pictured in an inverted microscope (Olympus, data not shown). For quantification of staining, 300 *μ*l of 10% (*v*/*v*) hexadecylpyridinium chloride monohydrate (CC, Sigma Aldrich, cat. no. 6004-24-06) was added to each well and placed at RT for 30 minutes shaking. Dye was removed from stain, and colour changes were seen in CC solution. 100 *μ*l/well solutions were transferred to a new 96-well plate and detected by spectrometry at 570 nm with FLUOstar Omega (BMG LABTECH, Offenburg, DE).

### 2.6. Morphological Examination of Cell Sheet and Histology

oADSCs from P1 were seeded (4000 cells/cm^2^ concentration) in a T75 flask and OIM as previously described. Cells were cultured for 14 days before the cell sheet was removed by cell scraper and washed two times in PBS and fixed in 4% paraformaldehyde. Paraffin-embedded histological analysis was made by section cuts to the cell sheet to a thickness of 4 *μ*m, deparaffinized, rehydrated, and stained with hematoxylin and eosin (H&E; DAKO-Aldrich, Denmark). Visualization pictures of cell sheet were captured with stereological software (newCAST™, Visiopharm, Denmark).

### 2.7. Statistical Analysis of Alizarin Red Quantification

Statistical analyses were performed using GraphPad Prism (version 7.03 software, La Jolla, CA). For normality, the Shapiro-Wilk test was performed. As data follow Gaussian distribution, ANOVA was made comparing the 4 test groups to the basic medium and OIM groups. Welch *t*-test was made comparing each group to the basic medium pairwise and OIM groups to reduce the risk of type I errors. Statistically significant values were defined as *p* < 0.05. Data is presented as the mean and standard deviation (SD).

## 3. Results

### 3.1. Characterization of oADSCs

The cells isolated from adipose tissue were plastic-adherent and had an average of 3.1% (range: 2.4%–3.9%) cells forming colonies from the isolated SVF after 12 days. No significant difference was seen between seeding density groups of 1·10^4^ cells and 1·10^3^ cells. Seeding density group of 1·10^5^ cells was left out due to overcolonization and impossible reliable counting. The average of 3.1% from the two lower seeding groups met the minimal criteria of more than 1% CFU proposed by Bourin et al. [11]. Seeding density of 1·10^3^ cells in T75 flask was most reliable for counting and therefore appropriate for use onwards.

**Figure 2:**
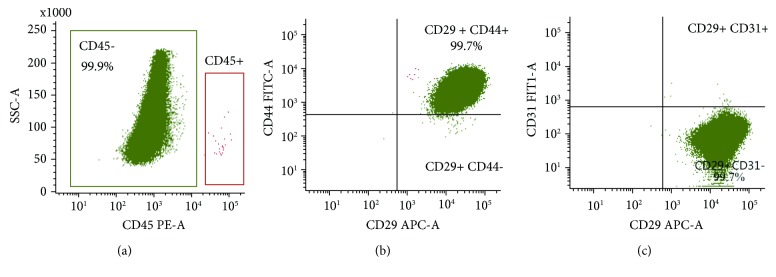
Immunophenotype of cells isolated from SVF conducted to be oADSCs. A gating of cells was based on FSC and SSC criteria, and the cells were subgated to only include single cells and only live cells were measured by fixable viability dye eFluor 506 staining and used subsequent analysis (data not shown). (a) CD45-negative cells were subgated to analyze the expression of (b) CD44 and CD29 and (c) CD31 and CD29. All quadrants were placed based on the isotype controls.

The ASC immunophenotype was confirmed as 99% of the gated live cells were positive for the surface markers CD29 and CD44 (two typical ASC surface markers) and less than 0.5% positive to CD31 (endothelial cells) and CD45 (leukocytes) (Figure 2). The cell culture thus met the minimal criteria for ASCs by having more than 80% CD29- and CD44-positive cells and less than 2% CD31- and CD45-positive cells.

Table 1 summarizes the minimal criteria set for immunophenotypic characterization of ASCs: a minimum of two positive and two negative surface markers as determined by Bourin et al. The flow cytometry of at least 250,000 cells analyzed in each sample shows that the cells express CD29 and CD44 but not CD31 and CD45.

### 3.2. Osteogenic Differentiation Optimization

**Figure 3:**
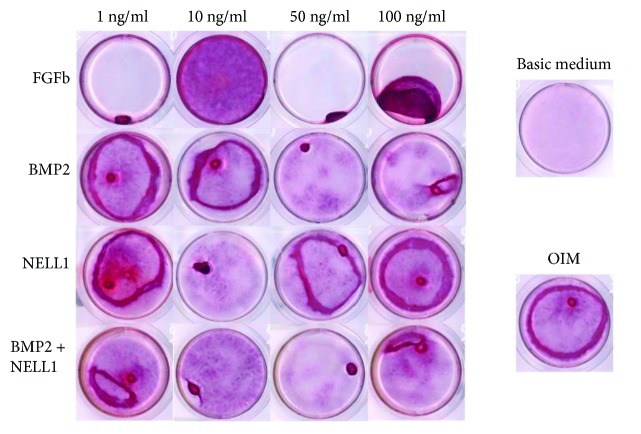
Alizarin red staining of oADSCs after a 14-day growth period in different growth mediums. Triplicates were made in all groups, and the most representative well from each group was chosen for the figure. The 4 × 4 square table is rhFGFb, rhBMP2, rhNELL1, and rhBMP2 plus rhNELL1 in different dosages with osteogenic induction medium (OIM). On the right the two controls: basic medium alone and OIM alone.

Results from 14-day growth tests in different osteogenic mediums were visualized by Alizarin red staining (ARS). Representative stains from each triplicate group are presented in Figure 3. During growth period, cell sheet was formed in the OIM groups. Detachment of cell sheet from well bottom was seen in most groups after 7–9 days (data not shown). Cell sheet folding in orb-formation and new colonization of well bottom was observed during the rest of the growth period visualized in Figure 3. No folding was seen in the FGFb 10 ng/ml group, and a better visualized ARS was observed after 14 days.

**Figure 4:**
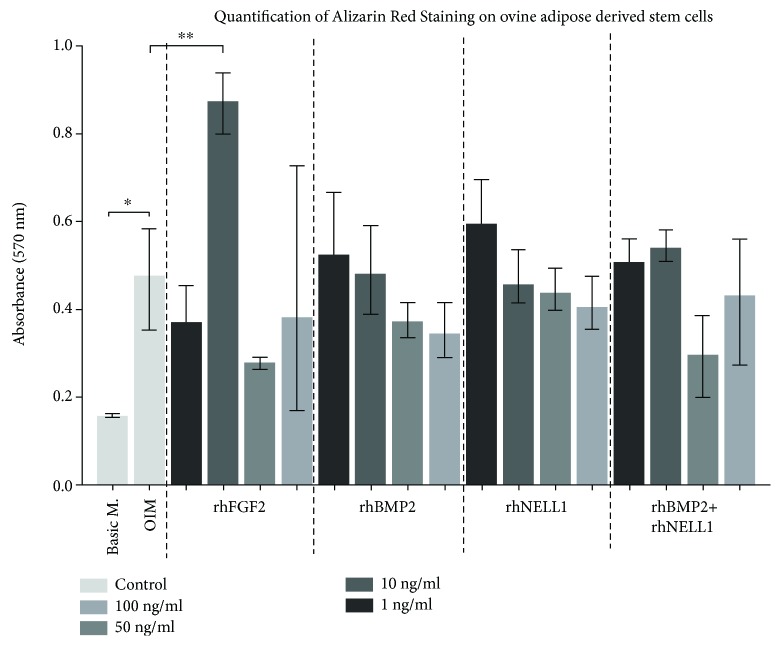
Quantification of the Alizarin red staining was made by hexadecylpyridinium chloride monohydrate (CC) after visualization. All wells were subtracted background from an average of 3 wells of 100 *μ*l CC. Unpaired ANOVA was made on the mean values with SD of all wells and compared with Basic M. (basic medium) and OIM alone by Welch *t*-test. ∗ indicates statistical significance (*p* < 0.05) between basic medium and OIM group. ^∗∗^ indicates statistical significance (*p* < 0.05) to OIM in a nonpaired *t*-test between the two control groups.

Significantly higher mineralization from quantification of ARS was seen among (12/17) groups containing only OIM or including growth factors compared to basic medium alone. (Only FGFb 10 ng/ml had significantly higher mineralization compared to OIM alone marked by the symbols ^∗∗^.) This was not evident in higher concentrations of FGFb (Figure 4). No significant dose response was observed when stimulated with FGFb, BMP2, NELL1, and a combination of BMP2 and NELL1.

## 4. Discussion

This current study showed that it is possible to isolate and verify oADSCs from ovine AT and optimize commitment to osteogenic differentiation with OIM when compared to standard growth medium after 14 days in vitro. Significant differentiation was shown to occur by adding FGFb to the OIM; however, this differentiation was not shown to be significantly affected by BMP2, NELL1, and a combination of BMP2 and NELL1. This supports our hypothesis that adding some bioactive factors to normal growth medium may significantly improve osteogenic differentiation and commitment to osteogenic lineage which may lead to an improved novo bone formation for later testing in vivo.

We aimed to harvest AT and isolate oADSCs from ovine and verify according to the minimal criteria for ASCs proposed by Bourin et al. [11]. Due to the low amount of available anti-ovine antibodies, only 3 primary anti-ovine were available. Anti-human CD29 (clone TS2/16) was used based on results from a basic local alignment search tool (BLAST) in the NCBI GenBank that showed sequence identity to the ovine form of integrin beta and had previously been used with success as antibody by Sanjurjo-Rodríguez et al. [37]. Both in terms of CFU assay and immunophenotypic characterization, this study succeeded in fulfilling the minimal criteria for ASCs isolated from ovine AT and proposes a feasible method for isolation of oADSCs from the lateral back of ovine.

A secondary aim of this study was to optimize the conditions for osteogenic differentiation and thereby select a better candidate for later examination in vivo. ARS and quantification were used to evaluate optimization by OIM and growth factors. We were able to show higher ARS and quantification after 14 days of culture in 12 out of 17 groups containing OIM (Figure 4) when compared to basic medium. The basic medium group represents our control cells, and the oADSCs in this group were treated as earlier cultures in vitro before implantation in vivo as was done in our previous study [13]. The OIM with FGFb 10 ng/ml showed significantly higher quantification than OIM alone. Both the results with OIM alone and FGFb 10 ng/ml suggested a stronger commitment to osteogenic cell lineage. To our knowledge, this study is the first to test FGFb on oADSC for osteogenic differentiation in vitro.

Cell sheet was formed in all groups that included OIM, and cell sheet detachment from well bottom and folding in orb formation was observed in 48 out of 51 wells. This may have limited visualization and might have also limited the quantification results due to poor cell growth the days after folding and cell necrosis. As a result of this, the precision regarding individual growth factors in different concentrations may be compromised and may not be included in the context of whether FGFb, BMP2, and NELL1 will boost commitment to osteogenic differentiation and thereby osteogenic lineage or not. Cell sheet formation is a response to L-ascorbic acid which makes cells deposit more collagen and thereby makes ECM [38]. ECM is considered a great carrier for mesenchymal stem cells [39] and causes problems when analysed by ARS due to in-between binding strength of the oADSCs compared to the plastic-adhered binding strength of the cells to well bottom. Nutrient necrosis of OIM for unknown reasons may also be a possibility for cell detachment. A visualization of the cell sheet was added as supplementary data, and a thick sheet with many cells in several layers surrounded by ECM is illustrated in Supplementary Figure 5. Recent steps toward optimizing ADSC sheet in canine in vitro were done by Kim et al. [40]. In a new study comparing ADMSC and ADMSC sheets, both groups included scaffolds in critical size defect dog model and showed significantly higher new bone formation after 12 weeks in the ADMSC sheet group [41]. The cell sheet folding may have limited the analysis of potential osteogenic lineage inducers of oADSCs, but it may propose a solution for controlling cells in future tissue engineering at focal region when implanted in critical size defects or in subcutaneous ectopic mice models.

In terms of immunophenotypic characterization, a clear limitation is the lack of more cross-reactive or primary antibodies for ovine to verify cells as adipose-derived mesenchymal stromal cells. This makes comparison between individual studies more difficult which is a problem summarized by Khan et al. [42].

A limitation to this study is the use of ARS as solo evaluation. Alkaline phosphatase activity has been shown to not express gen activity on ovine by Kalaszczynska et al. [43]. Preosteogenic markers like RUNX2, osteocalcin, type I collagen, and bone sialoprotein may be other options. Whether higher results at given time points yield more novo bone formation in vivo is unknown as many factors may influence the process from in vitro culture to in vivo bone formation [44]. In vivo experiments on small and large animal models must be done to show significant optimization.

Future in vivo investigations using small and large animals may be based on the optimal outcome from the current study and the cell sheet formation induced by OIM. Cell sheet may propose a solution for cell control with scaffold at focal region in animal models. The OIM may also have committed the oADSCs to osteogenic lineage. A hypothesis could be that changing from fetal bovine (FBS) to ovine serum may further optimize osteogenic differentiation of oADSCs.

We hypothesize that significantly more novo bone formation may be seen in vivo. This may lead to a more closely related human clinical relevance and can possibly make ASCs useful for future tissue engineering in clinical settings [45].

Whether results from in vitro can be translated into in vivo models remains to be seen. Sample sizes and changes to human samples along the way must be done to make these results translatable into the human clinical setting. To our knowledge, only limited research on osteogenic capacity between human and ovine has been investigated and differences may be expected [43].

## 5. Conclusion

This study successfully harvested AT from ovine and was verified for minimal criteria for ASCs which enables us to suggest that this is a feasible method for isolation of oADSCs. We were able to show significant effect of 10 ng/ml rhFGFb and OIM alone compared to basic growth medium but were not able to show dosage response with rhFGFb on osteogenic differentiation and commitment to osteogenic lineage. rhBMP2 and rhNELL1 added to OIM had no effect on osteogenic differentiation and commitment to osteogenic lineage based on ARS and quantification.

## Figures and Tables

**Table 1:** Immunophenotypic characterization.

Surface marker	ASCs criteria	Results from flow cytometry
CD29	Positive X > 80%	99.97% positive
CD44	Positive X > 80%	99.74% positive
CD45	Negative X < 2%	0.01% positive
CD31	Negative X < 2%	0.26% positive

## Data Availability

All data will be available from corresponding authors upon email if needed. Data is stored in 2 securely protected sites.

## Supplementary Materials

Supplementary materials Supplementary Figure 5 was added as supplementary material, and a detailed description of method can be found under Section 2.6. It shows hematoxylin and eosin (H&E) staining of a cell sheet after 14 days of growth. (A) Cell layer in 10x magnitude. (B) Cell layer in 40x. Cells are seen in many layers of the sheet with surrounding extracellular matrix.
