# Supplementary material for: Optimizing Osteogenic Differentiation of Ovine Adipose-Derived Stem Cells by Osteogenic Induction Medium and FGFb, BMP2, or NELL1 In Vitro
Source: Stem Cells Int. 2018 Sep 26;2018:9781393. doi: 10.1155/2018/9781393 (PMC6178511; doi:10.1155/2018/9781393)
Supplement: Supplementary materials — Supplementary Figure 5 was added as supplementary material, and a detailed description of method can be found under Section 2.6. It shows hematoxylin and eosin (H&E) staining of a cell sheet after 14 days of growth. (A) Cell layer in 10x magnitude. (B) Cell layer in 40x. Cells are seen in many layers of the sheet with surrounding extracellular matrix. [file 9781393.f1.pdf]

Supplementary materials

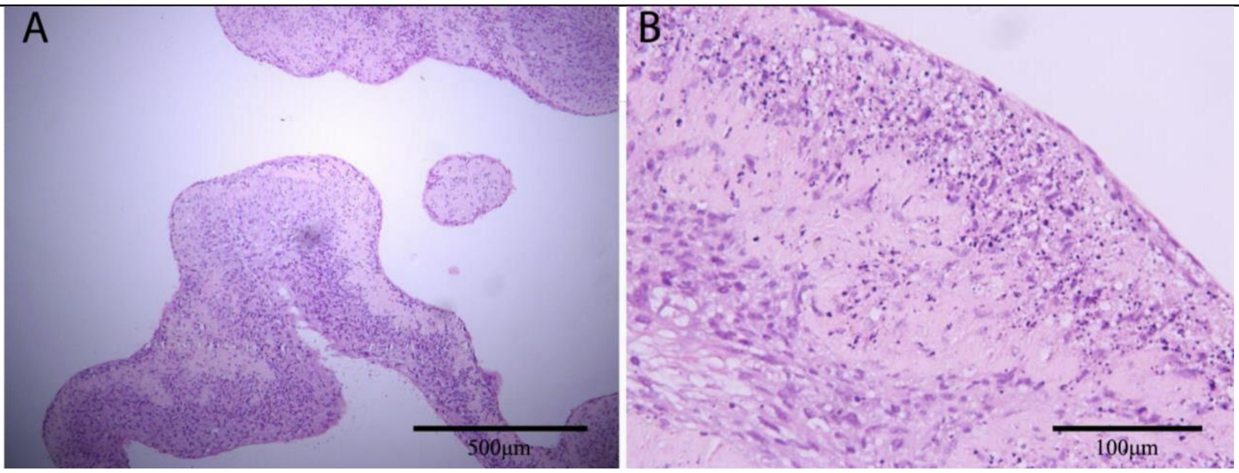

Figure 5: Hematoxylin and Eosin (H&E) staining of a cell sheet after 14 days of growth. A. shows cell layer in 10X magnitude. B. shows cell layer in 40X. Cells are seen in many layers of the sheet with surrounding extra cellular matrix.
